# The perceptual and mnemonic effects of ensemble representation on individual size representation

**DOI:** 10.3758/s13414-024-02963-x

**Published:** 2024-10-09

**Authors:** Yong Min Choi, Julie D. Golomb

**Affiliations:** Department of Psychology, The Ohio State University, Columbus, OH USA

**Keywords:** Visual perception, Perceptual organization, Attention

## Abstract

Our visual world consists of multiple objects, necessitating the identification of individual objects. Nevertheless, the representation of visual objects often exerts influence on each other. Even when we selectively attend to a subset of visual objects, the representations of surrounding items are encoded and influence the processing of the attended item(s). However, it remains unclear whether the effect of group ensemble representation on individual item representation occurs at the perceptual encoding phase, during the memory maintenance period, or both. Therefore, the current study conducted visual psychophysics experiments to investigate the contributions of perceptual and mnemonic bias on the observed effect of ensemble representation on individual size representation. Across five experiments, we found a consistent pattern of repulsive ensemble bias, such that the size of an individual target circle was consistently reported to be smaller than it actually was when presented alongside other circles with larger mean size, and vice versa. There was a perceptual component to the bias, but mnemonic factors also influenced its magnitude. Specifically, the repulsion bias was strongest with a short retention period (0–50 ms), then reduced within a second to a weaker magnitude that remained stable for a longer retention period (5,000 ms). Such patterns of results persisted when we facilitated the processing of ensemble representation by increasing the set size (Experiment 1B) or post-cueing the target circle so that attention was distributed across all items (Experiment 2B).

**Supplementary information:**

The online version contains supplementary material available at 10.3758/s13414-024-02963-x.

## Introduction

The visual system has a limited capacity to process multiple objects presented simultaneously (Luck & Vogel, 1997; Pylyshyn & Storm, 1988; Rensink et al., 1997). To efficiently process complex visual environments with given resources, the visual system prioritizes the processing of visual information more relevant to the current goal of behavior, known as *attention* (Carrasco, 2011; Chun et al., 2011). While we prioritize a subset of visual information, surrounding visual information does not go unnoticed. Instead, the representation of surrounding items is often still partially encoded and influences the processing of the task-relevant item (Brady & Alvarez, 2011; Choi et al., 2024; Chunharas et al., 2022; Gibson & Radner, 1937; Scotti et al., 2021).

The influence of simultaneous visual information on individual item representation is often found in tasks involving either perceptual or memory aspects, or both. There are some cases where the influence of surrounding visual information clearly occurs either at the early perceptual encoding phase (perceptual bias) or across the memory maintenance period (mnemonic bias). Visual illusions induced by surrounding visual stimuli are apparently perceptual since you can experience a distorted representation of individual items when you are looking directly at the stimuli. For example, the orientation of a grating at the center of the display appears to be biased away from the orientation of a surrounding grating with a different orientation (Tilt illusion; Gibson & Radner, 1937; O’Toole & Wenderoth, 1977), and a circle appears to be larger when surrounded by smaller circles, and vice versa (Ebbinghaus illusion; Roberts et al., 2005). On the other hand, there is evidence for individual item representations being biased actively during the memory maintenance period (Chunharas et al., 2022; Scotti et al., 2021). Using a delayed estimation task with varying memory retention periods, Scotti et al. (2021) and Chunharas et al. (2022) measured how much the representation of a remembered target item is biased relative to other memory stimuli that were presented simultaneously. They found a larger repulsion bias with longer memory delays. Moreover, the increased repulsion bias appears to be caused by active interactions between representations in memory, as Scotti et al. (2021) only found this effect when items needed to be actively maintained during the retention period, compared to performing a filler task during the retention period.

One case where it remains unclear whether the observed bias occurs at the perceptual encoding phase (perceptual bias) or during the memory retention period (memory bias) is the effect of group ensemble representation on individual item representation. When presented with multiple objects, group-level ensemble representation can influence the representation of individual items, referred to as the ensemble bias (Brady & Alvarez, 2011). For example, suppose you see a large flock of birds with similar colors, which makes it difficult to quickly discern the color of individual birds. When asked to report the color of a single bird in that flock, it may be advantageous to make use of higher-order group properties, such as the mean color of the flock, or the ensemble representation. Indeed, when presented with multiple objects, observers can rapidly extract summary statistics such as mean and variance of a group of items, known as *ensemble perception* (Alvarez, 2011; Chong & Treisman, 2003; Whitney & Yamanashi Leib, 2018). Complex visual information is encoded in multiple levels of abstraction, in which the representation of individual items and group-level summary statistics coexist hierarchically (Brady & Alvarez, 2011; Hochstein & Ahissar, 2002). This can lead to a biased representation of individual items, such as attraction bias toward the group average or repulsion bias away from the group average. Indeed, the attractive ensemble bias toward group-level visual information was found across multiple feature domains: size (Brady & Alvarez, 2011; Corbett, 2017; Yildirim et al., 2018), orientation (Utochkin & Brady, 2020), color (Son et al., 2020), facial emotion (Griffiths et al., 2018; Corbin & Crawford, 2018), and image valence (Alwis & Haberman, 2020).

To our knowledge, prior studies have not attempted to explicitly dissociate the effects of perceptual and mnemonic processes on ensemble bias. Part of the reason why many of these previous studies could not distinguish the contribution of perceptual and mnemonic bias is because they used a delayed response under the continuous adjustment report paradigm to measure the ensemble bias (Brady & Alvarez, 2011; Hseih et al., 2021, Utochkin & Brady, 2020). When asked to report the feature of the target item by adjusting the feature of the probe item, the response is made after a delay. In this case, the estimated ensemble bias could be influenced by both perceptual and mnemonic factors. Other studies have varied the temporal sequence of the stimuli, for example by presenting the probe circle either before or after the RSVP sequence of individual circles (Corbett & Oriet, 2011), but were not designed to measure the effect of ensemble representation on individual item representation, or the underlying sources (i.e., perceptual or mnemonic). Studies investigating temporal dynamics of ensemble encoding mainly focused on the encoding of ensemble representation from sequentially presented stimuli (Chong & Treisman, 2005) and how they are constrained by a limited temporal window (Attarha et al., 2016).

Here, we conducted a series of visual psychophysics experiments to investigate the source of ensemble bias for an individual target (i.e., attended) item’s size. In Experiment 1, we asked whether the ensemble bias occurs at the time of perceptual encoding (perceptual bias), or develops during the memory retention period (memory bias), by comparing the effect of group average on individual size representation between two conditions: one with limited involvement of mnemonic processing (the *perceptual bias condition; P*) and one with the involvement of both perceptual and mnemonic processing (the *perceptual* + *mnemonic bias; PM*). If the ensemble bias is mnemonic, we expected to find bias in the *PM*, but not in the *P condition*. In contrast, if the ensemble bias is mostly perceptual, we expected to find a comparable bias between the* P* and *PM conditions*. For preview, our results showed strong repulsion bias in the *P condition*, indicating the contribution of perceptual process on observed ensemble bias. Unexpectedly, we found reduced repulsion bias in the *PM condition* compared to the *P condition*, indicating an influence of the mnemonic process on ensemble bias, though in an unpredicted direction. To investigate the time course of the ensemble bias across memory retention periods, a series of experiments tested different retention periods ranging from 50 ms to 5,000 ms. We found the strongest repulsion bias with a short retention period (50 ms), which rapidly reduces within a second, and remains stable up to a longer retention period (5,000 ms). Importantly, the pattern of results did not change when we facilitated the processing of ensemble representation by increasing the set size (Experiment 1B) or post-cueing the target circle so that attention is distributed across all items (Experiment 2B).

## Experiment 1A

The goal of Experiment 1A was to examine the effect of ensemble representation on the size representation of a task-relevant item, specifically to investigate whether the observed ensemble bias is attributed to the perceptual, mnemonic process, or both. We asked subjects to perform a size comparison task between two white circles (target and probe circles) presented sequentially. The target circle was always presented along with three task-irrelevant black circles (reference set) that were either smaller or larger than the target circle, to induce contextual bias. Importantly, we manipulated when this target & reference set was presented relative to the probe circle. In the *perceptual* + *memory bias condition (PM*), we used a typical order from prior studies, where the target & reference set was presented at the beginning of the trial (study period) and the probe circle presented at the end (response period), such that the ensemble representation of the reference circles could influence the representation of the target circle through both perceptual and mnemonic processes. In contrast, in the *perceptual bias condition (P*), everything remained the same except the order was reversed, such that the probe circle was presented at the beginning of the trial and the target & reference set was presented at the end of the trial. Because the response in this condition was made immediately upon presentation of the target & reference display, any ensemble bias observed from the reference circles should mostly be attributable to the perceptual encoding phase, with limited involvement of mnemonic processing. By comparing the magnitude of ensemble bias resulting from these two conditions, we aimed to test whether the bias occurs at the perceptual encoding phase and/or during the memory retention period.

Methods.

### Participants

**Fig. 1:**
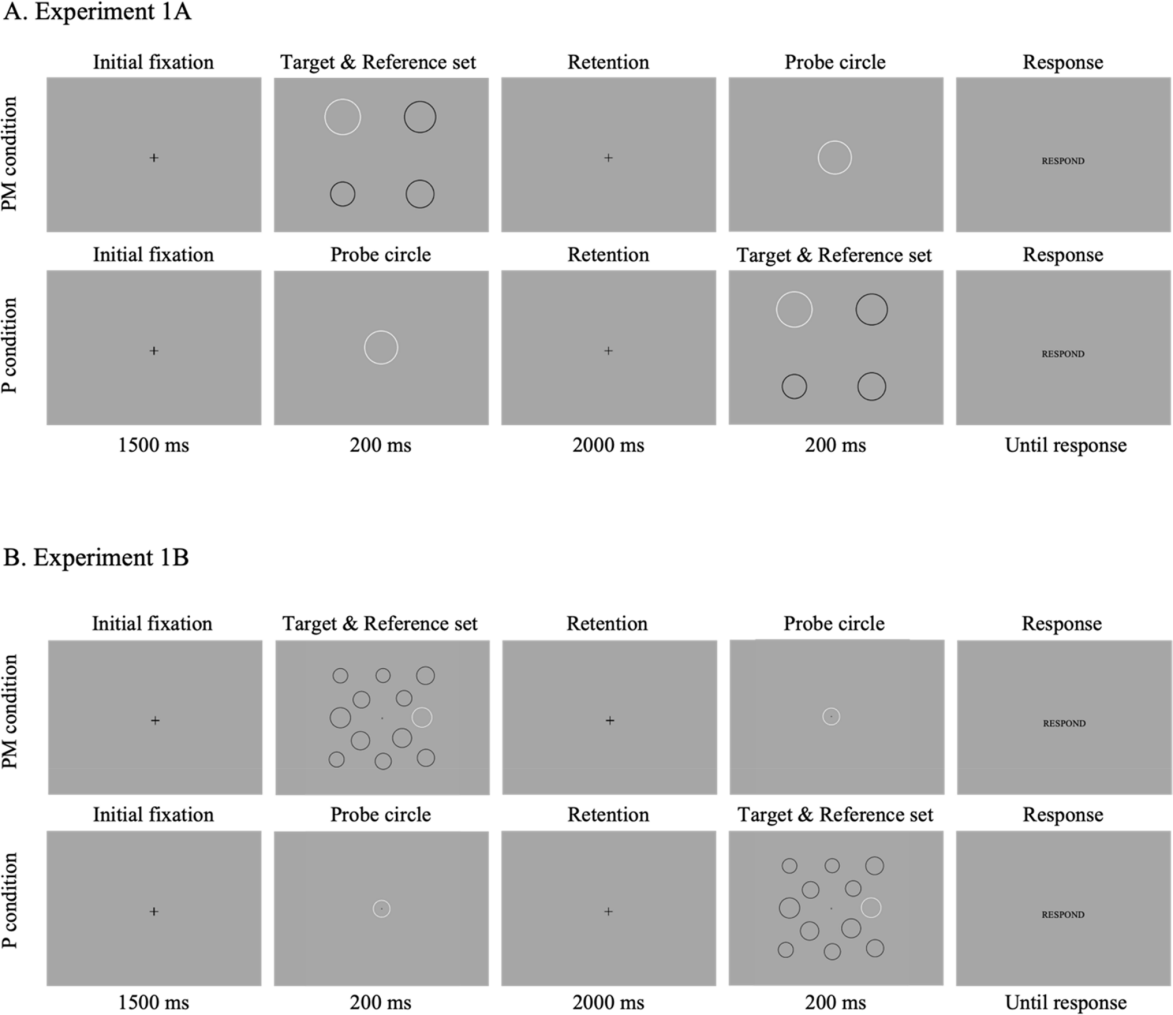
Trial sequences for Experiments 1A and 1B. Among two white circles presented for the size comparison task, the target circle was presented with task-irrelevant black circles with varying sizes (reference set) while the probe circle was always presented in isolation. The target & reference set was presented either earlier (the *PM condition*) or later (the *P condition*) than the probe circle. The reference set consisted of either three black circles in Experiment 1A (**A**), or 11 black circles in Experiment 1B (**B**)

A required sample size of N = 12 was calculated a priori based on a power analysis using G*Power version 3.1.9.6 (Faul et al., 2009). The power analysis was performed based on Griffith et al. (2018), where the judgments of the intensity of happy and angry expressions for individual faces are biased toward the average expression intensity of a group (*t*(23) = 5.83, *p* < 0.001, *d* = 1.19); although this study differed in the stimuli being compared, it was selected due to the similarity in stimulus configuration, task, and response mode (two-alternative forced-choice (2AFC) task). Given a significance criterion (*α*) of 0.05 and a power of 0.95, the power analysis yielded a required sample size of 12. We also set subject inclusion/exclusion criteria in advance, such that data obtained from a subject would be considered valid for inclusion if they completed the online-based experiment as instructed with more than 60% accuracy. Thirteen students (aged 18–20 years; nine women, four men, none non-binary) with normal or corrected-to-normal vision were recruited from the Ohio State University, with one participant excluded from the analysis due to an accuracy of less than 60% (see Online Supplementary Material (OSM) Fig. 1A). All Experiments were approved by the Ohio State University Behavioral and Social Sciences Institutional Review Board and every subject received either course credit or monetary compensation for participation.

### Stimuli and procedures

All stimuli were generated using MATLAB (The MathWorks, Natick, MA, USA) and configured as an online-based experiment using the Gorilla Experiment Builder (www.gorilla.sc; Anwyl-Irvine et al., 2019). On each trial, subjects were asked to compare two white circles (i.e., target and probe circles) presented sequentially, and report whether the one presented first or second was larger. One of the white circles (the probe circle*)* was always presented in isolation at the center of the display, while the other (the target circle*)* was presented together with three task-irrelevant black circles (the reference set) of varying sizes to induce ensemble bias. The target & reference set was either presented earlier (the *perceptual* + *memory bias condition; PM*) or later (the *perceptual bias condition; P*) than the probe circle.

As shown in Fig. 1A, each trial began with an initial fixation display (1,500 ms), followed by the first stimulus display – either the probe circle (*P condition*) or target & reference set (*PM condition*) – for 200 ms. After a 2,000-ms blank display, the second stimulus display appeared for 200 ms. As soon as the second stimulus disappeared, subjects responded whether the first or second white circle was larger by pressing either “F” or “J” on their keyboard as fast and accurately as possible.

The size of every circle presented during Experiment 1A was chosen from a set of 19 sizes (Table 1). The 19 sizes were calculated in pixels to maintain an 8% increment between adjacent sizes after converting the physical area into the perceived area through a power function with an exponent of 0.76 (Teghtsoonian, 1965). Stimuli were generated and saved as an image to be presented in a web environment. The probe circle was located at the center of an image. The target & reference set image included one white circle and three black circles located at each vertex of a right square with 300 pixels in width and height. The size of the white target circle was randomly selected between the seventh and 13th sizes in Table 1 (gray cells). The size of the white probe circle was selected so that it varied relative to the size of the target circle at one of seven different levels (-3, -2, -1, 0, 1, 2, and 3), to fit a psychometric function. The sizes of the black reference circles were selected as follows: In the *relatively small size (rel-small) condition*, the target circle was smaller than the three reference circles, which were chosen with two-step size increments in Table 1 (+ 2, + 4, and + 6 relative to the target size). In the *relatively large size (rel-large) condition*, the target circle was larger than the three reference circles (-2, -4, and -6 relative to the target size). For example, if the target circle on a given trial was Size8, the task-irrelevant reference circles would be Size10, Size12, and Size14 for the *rel-small condition*, or Size6, Size4, and Size2 for the *rel-large condition*. In either condition, the probe circle for the target comparison task in this example would be chosen between Size5 and Size11.

**Table 1:** Nineteen pre-determined circle sizes in radius (pixel)

Size	1st	2nd	3rd	4th	5th	6th	7th	8th	9th	10th	11th	12th	13th	14th	15th	16th	17th	18th	19th
Radius (pixel)	46	48	51	53	56	59	62	65	69	72	76	80	84	89	93	98	103	108	114

To summarize, Experiment 1A consisted of four conditions of interest: two bias conditions (*PM & M*) and two relative size conditions (*rel-small* and *rel-large*). For each of these conditions, we fit a psychometric function based on seven probe size conditions (-3, -2, -1, 0, 1, 2, and 3 relative to the target size). During the main session, each of these 28 conditions was repeated 28 times, resulting in 784 trials in total per participant. The trials were divided into 16 blocks with 49 trials each, and participants were allowed to take breaks as much as needed between each block. Feedback was provided only for 16 practice trials performed before the main session.

### Analysis

To assess whether subjects met the inclusion criteria (performance greater than 60% accuracy), we calculated accuracy after excluding trials in which the size of the test and target circle was identical. For the main analysis, we calculated the probability of reporting the target circle as larger than the probe circle as a function of all seven relative probe sizes, separately for the four main conditions. To quantify ensemble bias, data were fitted to a psychometric function. Specifically, we used the MATLAB Palamedes toolbox (Version 1.10.8; Prins & Kingdom, 2018) to fit the psychometric function with a hierarchical Bayesian approach. In the model, there were four parameters: location (α), slope (β), guess rate (γ), and lapse rate (δ). The location (α) parameter estimates the location of the psychometric function along the x-axis (the size of the probe circle required to be judged as identical to the size of the target circle). The slope (β) parameter estimates steepness of the psychometric function, and the lapse (δ) and guess rate (γ) parameters capture the upper and lower asymptotes of the psychometric function, respectively. The location (α) and slope (β) parameters were fit for each subject and each condition, whereas the guess rate (γ) and lapse rate (δ) parameters were only fit at the subject-level, since they reflect intrinsic response biases.

The key measure we assessed was the estimated location parameter (α) of the psychometric function, which indicates the size of the probe circle required to be judged as identical to the size of the target circle. Therefore, if the ensemble mean (mean size of the task-irrelevant reference set) influences the size representation of the target circle, we expected to see a shift in psychometric function between the *rel-small* and *rel-large* conditions.

The shift between conditions was examined by comparing group-level estimates of the location parameter between the *rel-small* and rel-*large size conditions*. For each bias condition (*PM* and *M*), we calculated ensemble bias by subtracting the posterior distribution of the location parameter of the *rel-large condition* from the *rel-small condition*. Then, we calculated the 89% highest density interval (HDI) to determine whether the deviation from zero is meaningful (McElreath, 2018). If the 89% HDI of the posterior difference distribution (α _rel-large_ - α _rel-small_) does not overlap with zero, we would conclude that there is evidence for biased size representation of the target circle relative to the mean size of the reference set.

## Results and discussion

**Fig. 2:**
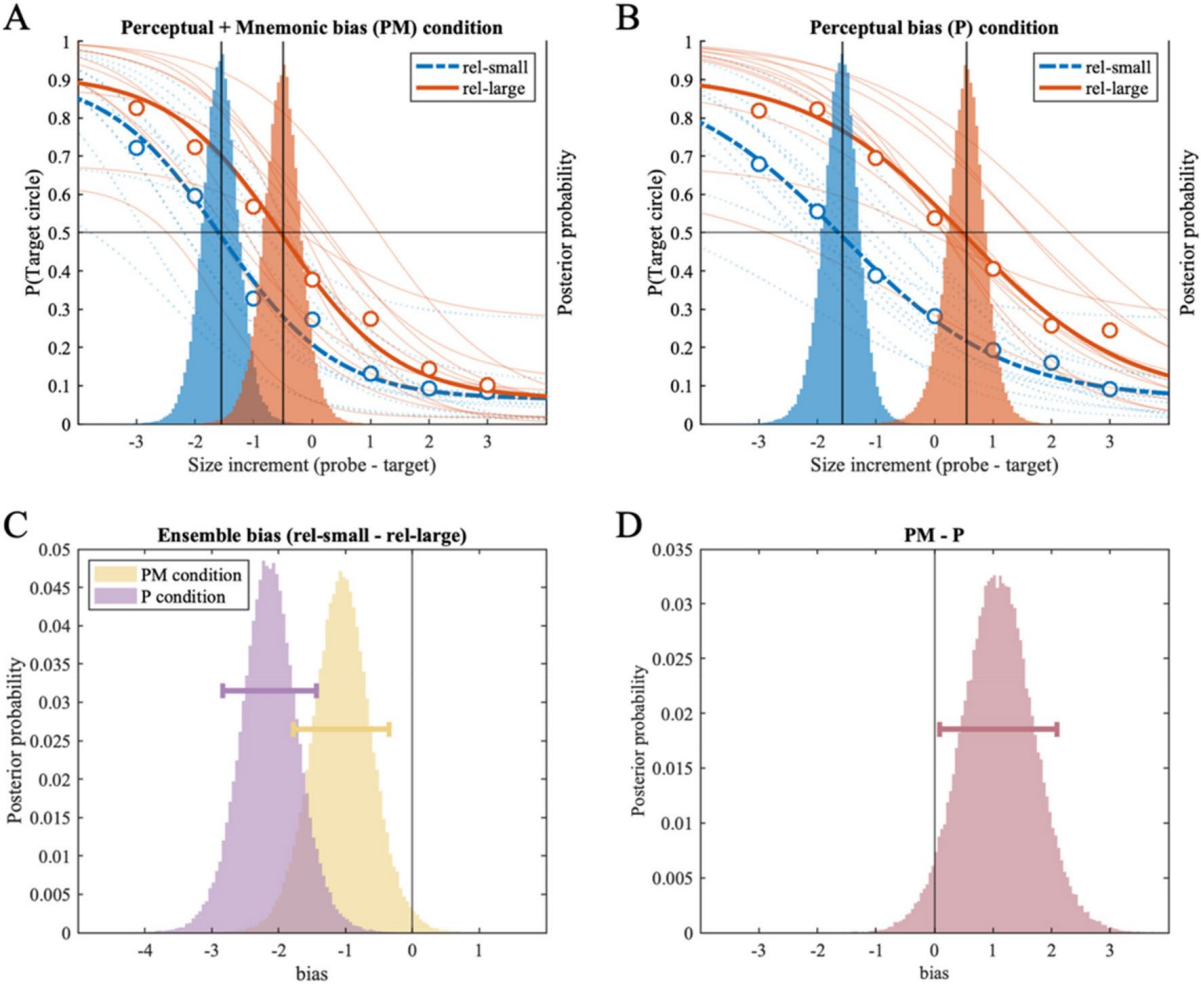
Results of Experiment 1A. Two panels on the top (**A:** the *Perceptual* + *Mnemonic bias condition*, **B:** the *Perceptual bias condition*) show fitted psychometric functions on the probability of reporting the target circle as larger (y-axis), as a function of size increment of the probe circle compared to the target circle (x-axis). Size increments on the x-axis correspond to the discrete set of 19 stimulus sizes used in the experiment; each increment is an 8% increase in perceived size (see *Method* section and Table 1). Group-level (thick lines) and individual-level (thin lines) psychometric functions are plotted for the *rel-small* (dotted line) and *rel-large* (solid) conditions; corresponding with the y-axis on the left. Group-level posterior distributions of location parameter (α) are plotted as a histogram behind the psychometric functions, referenced with the y-axis on the right. (**C**) Histograms showing the effect of ensemble representation on individual size representation in the *PM* and *P conditions*, respectively. Each histogram is generated by subtracting the location posterior distribution of the rel-large condition from that of the rel-small condition from A and B plots above; negative values indicate repulsion bias away from the ensemble mean size. (**D**) Histogram showing the difference between the distributions of ensemble bias in the *PM condition* and the *P condition*, indicating changes in ensemble bias with additional memory delay in the *PM condition*. Horizontal error bars indicate the 89% highest density interval

Figures 2A and 2B show the subject-level (thin lines) and group-level (thick lines) psychometric functions fitted to the *PM condition* (Fig. 2A) and the *P condition* (Fig. 2B). Each panel contains psychometric functions fitted to the *rel-small* and *rel-large size conditions,* where the task-relevant target circle was either smaller or larger than the task-irrelevant reference set. The x-axis indicates the size of the probe circle relative to the target circle (-3, -2, -1, 0, 1, 2, 3). The probability of reporting the target circle as larger is shown on the left side of the y-axis for the psychometric function and each data point (circular dots). In addition, the group-level posterior density distributions of the location parameter (α) of psychometric functions are overlaid on the same panel, corresponding to the right side of the y-axis. Note that intersections between the psychometric functions and a solid horizontal line, indicating the probability of 0.5, align with the locations of the peaks of posterior density distributions (solid vertical lines).

Visually, in *both* the *PM* (Fig. 2A) and the *P conditions* (Fig. 2B), the psychometric function of the *rel-large condition* is shifted rightward (or upward) compared to the *rel-small condition,* and posterior distribution of the location parameter is shifted accordingly. This indicates that the size of a target circle was more likely to be judged as larger when presented with the smaller reference set (the *rel-large condition*), compared to when presented with the larger reference set (the *rel-small condition*). This indicates ensemble bias in the form of repulsive bias away from the mean size of multiple circles. To quantitatively investigate the repulsion bias, we subtracted the posterior distribution of location estimates of the *rel-large condition* from that of the *rel-small condition* (Fig. 2C). In the *PM condition*, we found strong repulsion bias (MAP = -1.07, 89% HDI [- 1.78 -0.35]), with 98.68% of posterior samples smaller than zero. In the *P condition*, we found an even stronger repulsion bias (MAP = -2.18, 89% HDI [-2.83 -1.43]), with 99.99% of posterior samples located below zero. These results clearly show a biased representation of individual size away from the size of the group's mean size in both *P* and *PM condition*s.

Lastly, to compare the two bias conditions (*P* and *PM*), we obtained a distribution of difference between the ensemble bias found in the *P condition* from the *PM condition* (Fig. 2D). We found reduced repulsion bias in the *PM* compared to the *P condition*, indicated by a negatively shifted posterior distribution with 95.78% of posterior samples larger than zero (MAP = 1.00, 89% HDI [0.09 2.10]).

To summarize Experiment 1A, we found a robust effect of ensemble representation on individual size representation, in the form of repulsion bias in both the *PM* and* P conditions.* This suggests that the observed ensemble bias has a strong perceptual component. Unexpectedly, the repulsion bias was larger in the *P condition* compared to the *PM condition*. One possible explanation is that the repulsion bias is maximal during perception (the target’s size is perceived away from the group mean size during stimulus presentation), and then reduces such that the representation becomes more veridical in memory. Alternatively, it is possible that the reduced repulsion bias in the PM condition was driven by a difference in task design and presentation order for the* P* and *PM conditions*. In a series of following experiments, we first confirm (1) if strong perceptual bias persists in the *P condition* with a larger reference set size (Experiment 1B), and then further investigate (2) how mnemonic components influence ensemble bias by comparing PM conditions with varying memory delays (Experiments 2 and 3).

## Experiment 1B

Experiment 1A showed a robust repulsion bias away from the size of surrounding circles by presenting one target circle together with a task-irrelevant reference set of three black circles. In Experiment 1B, we asked whether the findings would replicate with a larger task-irrelevant set size consisting of 12 circles. According to previous studies, larger set sizes lead to better extraction of summary statistics such as mean size (Robitaille & Harris, 2011), orientation (Chetverikov et al., 2017; Robitaille & Harris, 2011), or color (Maule & Franklin, 2015). With a more precise representation of surrounding information, the mean size of the surrounding circles may exert a stronger influence on the size of individual circles (Utochkin & Brady, 2020).

Methods.

### Participants

The required sample size for Experiment 1B was N = 12, following that of Experiment 1A. Each subject was considered valid if they completed the experiment as instructed with an accuracy higher than 60%. Twelve students (aged 18–20 years; eight women, four men, none non-binary) with normal or corrected-to-normal vision were recruited from the Ohio State University. All subjects showed accuracy higher than 60% (OSM Fig. 1B).

### Stimuli and procedures

Unlike Experiment 1A, Experiment 1B was conducted in person. All stimuli were generated and presented using MATLAB with the Psychophysics Toolbox (Version 3 extension; Brainard, 1997; Kleiner et al., 2007; Pelli, 1997). Experiment stimuli were presented on a gray background using a 21-in. flat-screen LCD monitor with a refresh rate of 240 Hz and a screen resolution of 1,920 $\times$ 1,080 pixels. Subjects were distanced 63 cm from the monitor using a headrest in a dark room (37 pixels per visual degree).

While the trial procedure was identical to Experiment 1A, the number and configuration of circles presented for the target & reference set were different (Fig. 1B). Here, the target circle was always presented with 11 task-irrelevant black circles (reference set). The 12 circles in the target & reference set were located at 12 pre-determined locations, plus random jitter sampled from a normal distribution in orthogonal directions (M = 0, SD = 10 pixels). Four inner circles were placed at the four corners of an 8 degrees right square, and eight outer circles were placed at the four corners and midpoint of each side of a 16 degrees right square (Fig. 1B). The white target circle could appear in any of the 12 locations. Every circle was drawn with a 4-pixel solid line without filled color. The target & reference set was either presented first (*PM*: Fig. 1B, top row) or second (*P*: Fig. 1B, bottom row) to test whether ensemble bias occurs at the perceptual encoding phase, or during the memory maintenance period.

The sizes of the 12 circles were determined at the beginning of each trial as follows. Note that unlike Experiment 1A, which used pre-generated stimuli from a limited set of sizes for online presentation, Experiment 1B was conducted in person without a pre-determined set of sizes. First, the radius of the smallest circle among the target & reference set was randomly chosen between 1.2 and 1.8 visual degrees. The other 11 sizes were generated to have a constant increase rate of 5% after converting into the perceived area using a power function with an exponent of 0.76 (Teghtsoonian, 1965). The target circle was set as the second-to-largest size of the 12 circles in half of the trials (the *rel-large condition*) or the second-to-smallest in the other half (the *rel-small condition*). As in Experiment 1A, the size of the probe circle was selected so that it varied relative to the size of the target circle at one of seven different levels (-3, -2, -1, 0, 1, 2, & 3) to allow fitting a psychometric function. The seven possible probe sizes were calculated to have a constant increase rate of 8% on the perceived scale (Teghtsoonian, 1965).

To summarize, two bias conditions (*PM* and *M*), two relative size conditions (the *rel-small* and *rel-large conditions*), and seven probe size conditions (-3, -2, -1, 0, 1, 2, and 3) were repeated 24 times, resulting in 672 trials in total. 672 trials were divided into 12 blocks with 56 trials, and participants were allowed to take breaks as much as needed between each block. Before the main session, participants performed 16 practice trials with visual feedback.

### Analysis

The analysis of Experiment 1B was identical to that of Experiment 1A.

## Results and discussion

**Fig. 3:**
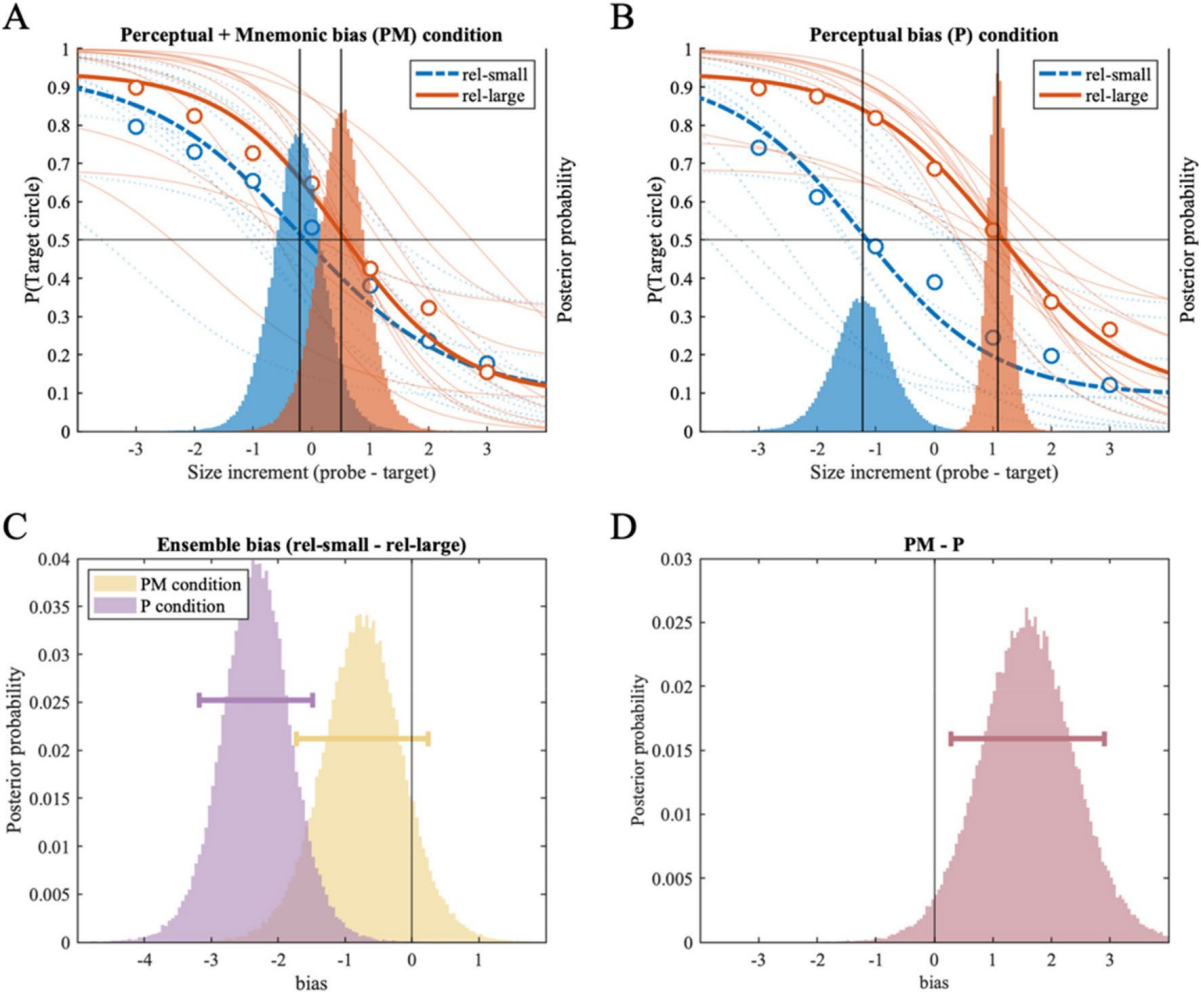
Results of Experiment 1B. Two panels on the top (**A:** the *Perceptual* + *Mnemonic bias condition,* **B:** the *Perceptual bias condition*) show fitted psychometric functions on the probability of reporting the target circle as larger (y-axis), as a function of size increment of the probe circle compared to the target circle (x-axis). Size increments on the x-axis correspond to the discrete set of 19 stimulus sizes used in the experiment; each increment is an 8% increase in perceived size (see *Method* section and Table 1). Group-level (thick lines) and individual-level (thin lines) psychometric functions are plotted for the *rel-small* (dotted line) and *rel-large* (solid) conditions; corresponding with the y-axis on the left. Group-level posterior distributions of location parameter (α) are plotted as a histogram behind the psychometric functions, referenced with the y-axis on the right. (**C**) Histograms showing the effect of ensemble representation on individual size representation in the *PM* and *P conditions*, respectively. Each histogram is generated by subtracting the location posterior distribution of the rel-large condition from that of the rel-small condition from A and B plots above; negative values indicate repulsion bias away from the ensemble mean size. (**D**) Histogram showing the difference between the distributions of ensemble bias in the *PM condition* and the *P condition*, indicating changes in ensemble bias with additional memory delay in the *PM condition*. Horizontal error bars indicate the 89% highest density interval

Overall, the results of Experiment 1B replicated Experiment 1A, with a larger reference set size to facilitate the processing of summary statistics of multiple items. As shown in Fig. 3, the psychometric function of the *rel-large condition* is shifted to the right compared to the *rel-small condition* in *both* the *PM* (Fig. 3A) and the *P condition* (Fig. 3B). This rightward shift indicates an overestimated size representation of the target circle when presented with a task-irrelevant reference set of mostly smaller circles (the *rel-large condition*), compared to when presented with a reference set of mostly larger circles (the *rel-small condition*). When quantitatively investigating the difference between the two posterior location distributions (Fig. 3C), we found a repulsion bias in the *P condition* with 99.96% of posterior samples located below zero (MAP = -2.32, 89% HDI [-3.18 -1.48]). In the *PM condition*, however, we found no reliable repulsion bias (MAP = -0.76, 89% HDI [-1.73 0.24]). Nevertheless, 88.85% of posterior samples were smaller than zero, providing weak evidence for the repulsion bias away from reference circles. When we subtracted measured bias in the *P condition* from the *PM condition*, we found decreased repulsion bias in the *PM condition* compared to the *P condition* (MAP = 1.61, 89% HDI [0.29 2.91]), with 97.29% of posterior samples larger than zero.

## Experiment 2A

In Experiments 1A and 1B, we found a strong repulsion bias regardless of set size. The repulsion bias was robust in the *P condition*, but somewhat diminished in the *PM condition*. Although we found a smaller repulsion bias in the *PM condition*, it remains unclear whether the reduction is due to mnemonic processing during the 2,000-ms memory delay, or due to a qualitatively different design between the* P* and *PM conditions*. To test whether and how the ensemble bias changes over time across the memory retention period, Experiment 2 tested two *PM* conditions with different memory delays (1,000 ms and 3,000 ms). The duration of these two retention periods was chosen based on Scotti et al. (2021), where they found a repulsion bias between the color of two objects with a 3-s delay condition, but not with a 1-s delay condition, indicating active interaction between working memory contents during the retention period. In Experiment 2A, we used an identical *PM* design to that in Experiment 1A, but with either 1,000-ms or 3,000-ms memory delays instead of the fixed 2,000-ms delay.

Methods.

### Participants

The sample size (N = 12) and criteria for subject validity were identical to Experiment 1. We recruited participants until the number of valid subjects matched the required sample size. Fifteen students (aged 18–20 years; four women, 11 men, none non-binary) from the Ohio State University participated in Experiment 2A, and 12 subjects were counted as valid (OSM Fig. 1C). Every participant reported having a normal or corrected-to-normal vision.

### Stimuli and procedures

**Fig. 4:**
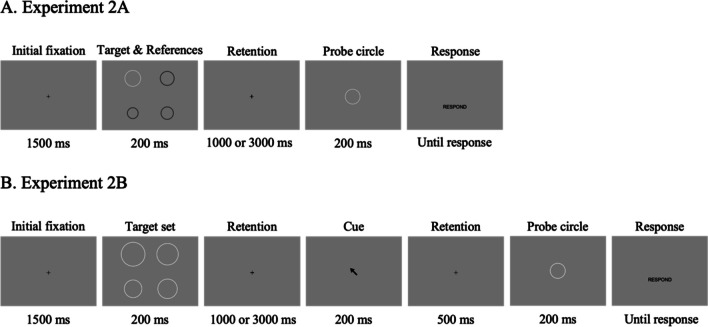
Trial sequences for Experiments 2A and 2B. (**A**) Trial sequence of Experiment 2A was adopted from the *PM condition* in Experiments 1A and 1B, except that the retention period varied between 1,000 ms and 3,000 ms. (**B**) In Experiment 2B, the target set consisted of four white circles, and one of them was cued afterward as a target circle that should be compared with the following probe circle. Therefore, the subject had to memorize the size of all four circles and used one of them for the size comparison task. The arrow cue was presented after either 1,000 ms or 3,000 ms, followed by a 500-ms blank period before the presentation of the second stimulus display

The stimuli and procedures of Experiment 2A were adopted from the *PM condition* in Experiment 1A. In every trial in Experiment 2A (Fig. 4A), the target & reference set had a set size of four circles, and was always presented at the beginning of the trial, with the probe circle presented at the end of the trial. Notably, we varied the memory retention interval between the target & reference set and the probe circle: half of the trials were *1,000-ms* and half were *3,000-ms conditions* (intermixed). Experiment 2A thus consisted of two retention period conditions (*1,000 ms* and *3,000 ms*), two relative target size conditions (*rel-small* and *rel-large*), and seven probe size conditions (-3, -2, -1, 0, 1, 2, and 3 relative to the size of the target circle, as before). Following 16 practice trials with feedback, each combination of conditions was repeated 28 times, resulting in 784 trials in total (28 conditions $\times$ 28 repetitions). The 784 trials were divided into 16 blocks with 49 trials, and participants were allowed to take breaks as much as needed between each block. Visual feedback was provided only for 16 practice trials.

### Analysis

The subject exclusion criterion and method to estimate parameters from the fitted psychometric function were identical to that of Experiment 1. However, in Experiment 2A, we focused on comparing two conditions with different memory delay periods (1,000-ms vs. 3,000-ms retention period conditions).

## Results and discussion

**Fig. 5:**
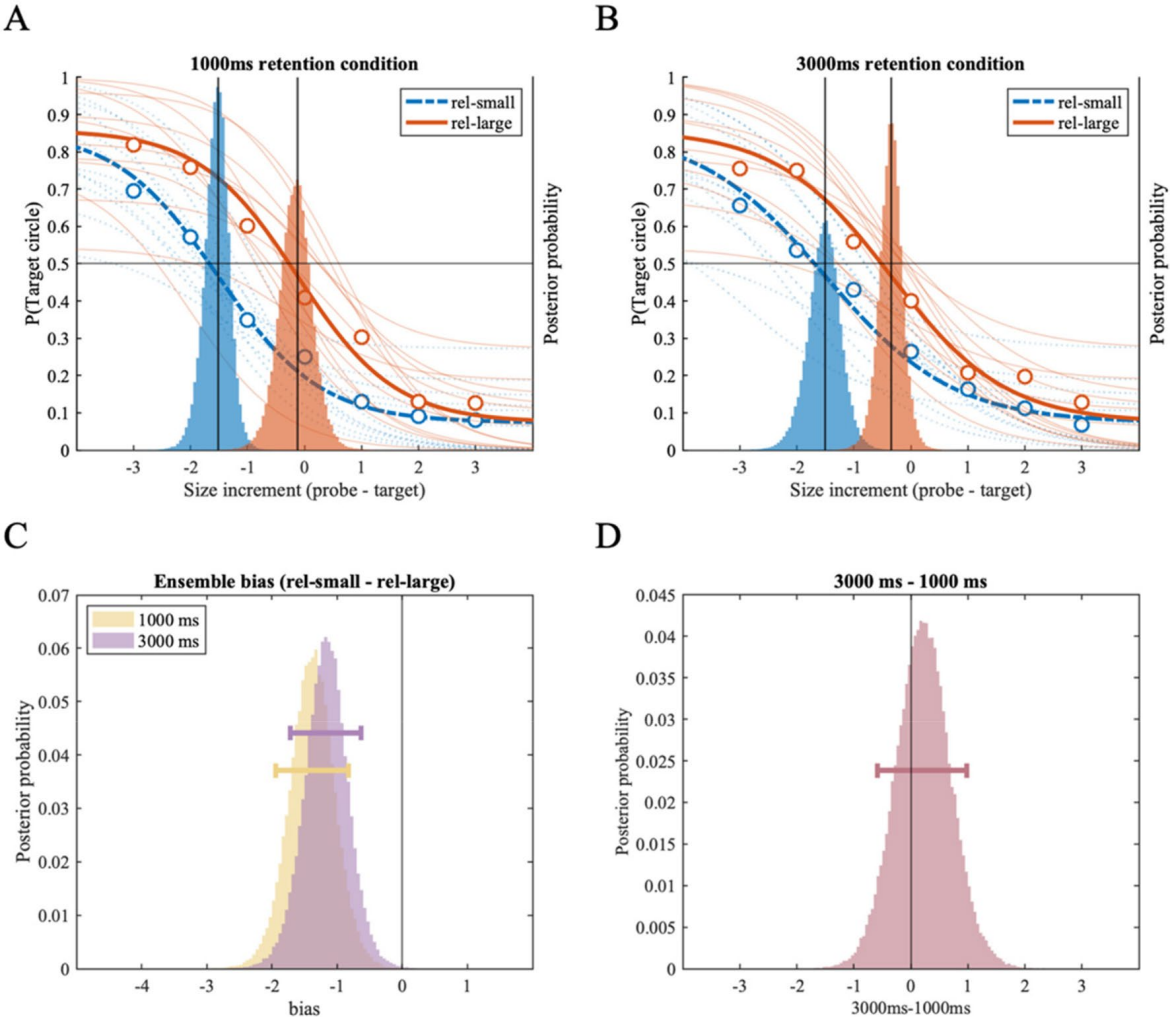
Results of Experiment 2A. Two panels on the top (**A:** the *1,000-ms retention period,* **B:** the *3,000-ms retention period*) show fitted psychometric functions on the probability of reporting the target circle as larger (y-axis), as a function of size increment of the probe circle compared to the target circle (x-axis). Size increments on the x-axis correspond to the discrete set of 19 stimulus sizes used in the experiment; each increment is an 8% increase in perceived size (see *Method* section and Table 1). Group-level (thick lines) and individual-level (thin lines) psychometric functions are plotted for the *rel-small* (dotted line) and *rel-large* (solid) conditions; corresponding with the y-axis on the left. Group-level posterior distributions of location parameter (α) are plotted as a histogram behind the psychometric functions, referenced with the y-axis on the right. (**C**) Histograms showing the effect of ensemble representation on individual size representation in *the 1,000 ms* and *3,000 ms conditions*, respectively. Each histogram is generated by subtracting the location posterior distribution of the *rel-large condition* from that of the *rel-small condition* from A and B plots above; negative values indicate repulsion bias away from the ensemble mean size. (**D**) Histogram showing the difference between the distributions of ensemble bias in the *1,000-ms condition* and the *3,000-ms condition*, indicating changes in ensemble bias with additional memory delay in the *3,000-ms condition*. Horizontal error bars indicate the 89% highest density interval

As shown in Figs. 5A and 5B, the psychometric function fitted to the *rel-large condition* was shifted to the right compared to that of the *rel-small condition*, in both delay conditions. The difference plots (Fig. 5C) revealed robust repulsion bias in both the *1,000-ms retention period condition*, with 99.97% of posterior samples smaller than zero (MAP = -1.35, 89% HDI [-1.94 -0.82]), and the *3,000-ms retention period condition,* with 99.89% of posterior samples smaller than zero (MAP = -1.17, 89% HDI [-1.72 -0.63]). More importantly, we subtracted the posterior distribution of the *1,000-ms* from that of the *3,000-ms retention period condition*, to test whether the ensemble bias changes between *1,000-ms* and *3,000-ms memory delay* (Fig. 5D). As shown in Fig. 5D, the posterior samples indicating the influence of the mnemonic process on ensemble bias were mostly centered around zero (MAP = 0.21, 89% HDI [-0.58 0.98]), with 67.38% of posterior samples above zero. The absence of a credible difference between the two retention period conditions suggests that the effect of group ensemble representation on individual size representation did not change within the 1,000-ms and 3,000-ms retention period window.

To summarize, while Experiment 2A replicated the repulsion bias found in preceding experiments, the magnitude of ensemble bias was not changed after the 3,000-ms retention period compared to the 1,000-ms retention period. Indeed, the magnitudes of bias found in the *1,000-ms* (MAP = -1.35) and the *3,000-ms retention period condition* (MAP = -1.17) were comparable to that of the *PM condition* in Experiment 1A with 2,000-ms retention period (MAP = -1.07); see *Meta-analyses* section for more across-experiment analyses.

## Experiment 2B

Experiment 2B was conducted to test the same question asked in Experiment 2A, while accounting for a potential limitation. In the preceding experiments, the target circle (white) was always perceptually distinct from the surrounding reference set (black). Using a distinctive color was intentional for our design and was especially critical in Experiment 1 so we could compare the *PM* and *P conditions*; the color cue helped subjects to identify the target circle at the moment they see the target & reference set, allowing the *P condition* to be minimally affected by the mnemonic process in Experiment 1. The same stimuli were used in Experiment 2A to manipulate retention period while maintaining the same exact design as in the *PM condition* in Experiment 1. However, using a distinct target color may have limited the effect of group ensemble representation on individual size representation. First, the target and reference circles may have been encoded as two different groups. Prior work has shown that the effect of ensemble representation on individual representation depends on how items can be perceptually grouped. For example, when circles of multiple colors were presented, the individual size representation of a given circle was biased toward the average size of the same color group (Brady & Alvarez, 2011), or other grouping cues (Corbett, 2017; Yildirim et al., 2018). Additionally, the use of fixed target and distractor colors may have facilitated a more focused mode of attention, whereas ensemble representations often tend to be better encoded under a more distributed mode of attention (Baek & Chong, 2020; Chong & Treisman, 2005).

While we found a reliable ensemble bias in the preceding experiments, it is possible that the magnitude and direction of the effect were limited by our design. Thereafter, we wanted to ensure that the lack of memory retention effect was generalizable to a more robust ensemble bias context. Previous studies have shown that the representation of individual orientation (Utochkin & Brady, 2020) or mean size of a subset of stimuli (Yildirim et al., 2018) relied more on the summary statistics of entire stimuli when the target was cued after stimuli presentation (with a post-cue) compared to when cued before presentation (pre-cue). Thus, in Experiment 2B, we repeated the same question as Experiment 2A – whether ensemble bias on individual size representation differs for 1,000-ms versus 3,000-ms memory delays – but modified our paradigm such that the target circle was not perceptually distinct from the reference circles and could only be identified with a subsequent post-cue.

Methods.

### Participants

We recruited participants until the number of valid subjects matched the required sample size (N = 12). The criteria for subject validity were identical to Experiment 1. Nineteen students (aged 18–22 years; 13 women, six men, none non-binary) from the Ohio State University participated in Experiment 2B and 12 subjects were counted as valid (OSM Fig. 1D).

### Stimuli and procedures

In Experiment 2B, the experiment design and stimuli were slightly changed from that of Experiment 2A. As shown in Fig. 4B, instead of a single white target circle and three black reference circles, we presented all four circles in white as a target set. Participants were instructed to encode and remember the size of all four items until the post-cue. The post-cue was an arrow presented at the center of the display for 200 ms pointing toward the task-relevant (target) item for the size comparison task. To manipulate the length of the retention period, the arrow cue was presented after either a *1,000-ms* or a *3,000-ms retention period*. Therefore, subjects had to remember the size of all four circles at first, and then compare the size of the post-cued target circle with the size of the probe circle presented afterward.

Two retention period conditions (*1,000-ms* and *3,000-ms conditions*), two relative size conditions (the *rel-small* and *rel-large size conditions*), and seven probe sizes relative to the target size (-3, -2, -1, 0, 1, 2, and 3) were repeated 28 times. A total of 784 main trials (28 conditions $\times$ 28 repetitions) were divided into 16 blocks with 49 trials, and every subject performed 16 practice trials before the main trials.

### Analysis

The analyses for Experiment 2B were identical to that of the preceding experiments.

## Results and discussion

**Fig. 6:**
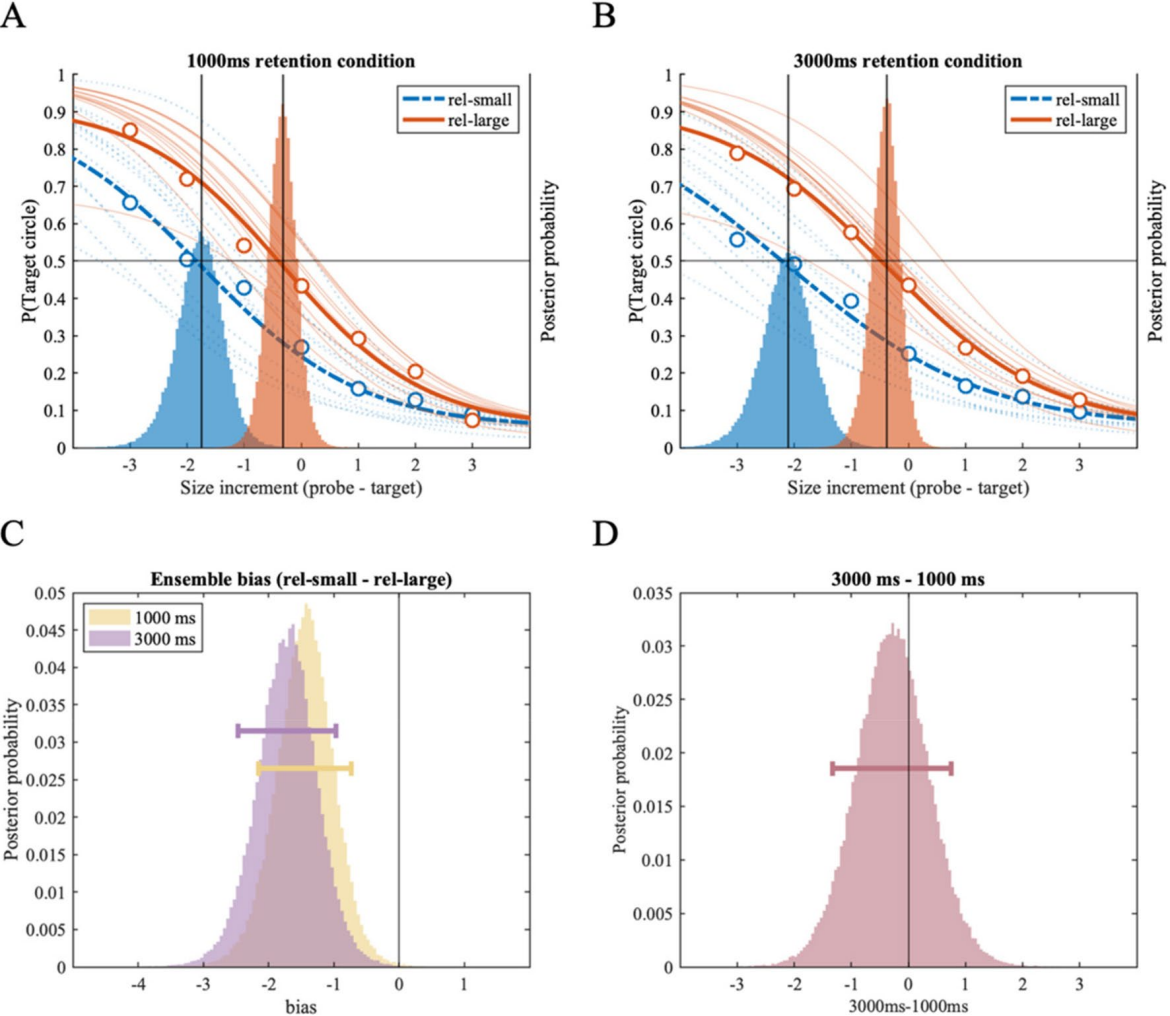
Results of Experiment 2B. Two panels on the top (**A:** the *1,000-ms retention period,* **B:** the *3,000-ms retention period*) show fitted psychometric functions on the probability of reporting the target circle as larger (y-axis), as a function of size increment of the probe circle compared to the target circle (x-axis). Size increments on the x-axis correspond to the discrete set of 19 stimulus sizes used in the experiment; each increment is an 8% increase in perceived size (see *Method* section and Table 1). Group-level (thick lines) and individual-level (thin lines) psychometric functions are plotted for the *rel-small* (dotted line) and *rel-large* (solid) conditions; corresponding with the y-axis on the left. Group-level posterior distributions of location parameter (α) are plotted as a histogram behind the psychometric functions, referenced with the y-axis on the right. (**C**) Histograms showing the effect of ensemble representation on individual size representation in the *1,000-ms* and *3,000-ms conditions*, respectively. Each histogram is generated by subtracting the location posterior distribution of the rel-large condition from that of the rel-small condition from A and B plots above; negative values indicate repulsion bias away from the ensemble mean size. (**D**) Histogram showing the difference between the distributions of ensemble bias in the *1,000-ms condition* and the *3,000-ms condition*, indicating changes in ensemble bias with additional memory delay in the *3,000-ms condition*. Horizontal error bars indicate the 89% highest density interval

The results of Experiment 2B, in which we compared ensemble bias between the *1,000-ms* and *3,000-ms retention period conditions* while facilitating the use of distributed attention by using a post-cue, closely mimicked Experiment 2A. First, we found strong repulsion bias across both the *1,000-ms* and *3,000-ms retention period conditions* (Fig. 6). Psychometric functions fitted to the *rel-large condition* were shifted to the right compared to that of the *rel-small condition* (Figs. 6A and 6B). The difference between the two posterior distributions of location parameter revealed robust repulsion bias in both the *1,000-ms retention period condition*, with 98.84% of posterior samples smaller than zero (MAP = -1.41, 89% HDI [-2.16 -0.74]), and the *3,000-ms retention period condition* with 99.94% of posterior samples smaller than zero (MAP = -1.65, 89% HDI [-2.47 -0.97]). As shown in Fig. 6D, the ensemble bias was not changed after the 3,000-ms retention period compared to the 1,000-ms retention period, indicated by HDI mostly centered around zero (MAP = -0.27, 89% HDI [-1.33 0.75]). We also conducted an additional, supplementary experiment using a continuous adjustment report paradigm (OSM Fig. 2; pre-registered at OSF: https://osf.io/vkx5h/). Using a similar distributed attention design to that in Experiment 2B, but with a continuous report paradigm, we replicated repulsion bias for both 1,000-ms and 3,000-ms memory retention periods.

To summarize, the results of Experiment 2B and the supplemental experiment suggest robust repulsion bias away from the group mean size regardless of the memory retention period, even when the experimental design does not facilitate selective processing of the target circle at encoding, but instead requires distributed attention toward a set of possible target circles.

## Experiment 3

The preceding experiments showed the representation of an individual circle consistently biased away from simultaneously presented circles (repulsion bias). Experiment 1 showed a smaller repulsion bias in the *PM bias condition* with a 2,000-ms memory delay compared to the *P condition* with no memory component, and Experiment 2 found a comparable repulsion bias between *PM conditions* with 1,000-ms and 3,000-ms memory delays.

The pattern of stronger bias in the *P condition* compared to any conditions with memory delay could suggest that the repulsion bias is maximal following initial encoding phase and reduced following an additional memory retention period. However, it is difficult to conclude this from these studies alone, because the experiment designs of the P and PM conditions are qualitatively different from each other (i.e., the *P condition* is not necessarily the same as a* 0-ms retention period PM condition*). Therefore, Experiment 3 was conducted to explicitly test this by having a memory retention (PM) condition with a negligible delay (only 50 ms). Additionally, we tested a *5,000-ms retention period condition* to test if there are additional effects of the mnemonic process at even longer delays.

Methods.

### Participants

We recruited participants until the number of valid subjects matched the required sample size (N = 12). The criteria for subject validity were identical to Experiment 1. Thirteen students (aged 18–19 years; 12 women, no men, one non-binary) from the Ohio State University participated, and 12 subjects were counted as valid (OSM Fig. 1E).

### Stimuli and procedures

The stimuli and procedure of Experiment 3 were identical to that of Experiment 2A (Fig. 4A), except that the probe circle was presented after a *50-ms* or *5,000-ms retention period*.

### Analysis

The analysis performed in Experiment 3 was identical to that of Experiment 2.

## Results and discussion

**Fig. 7:**
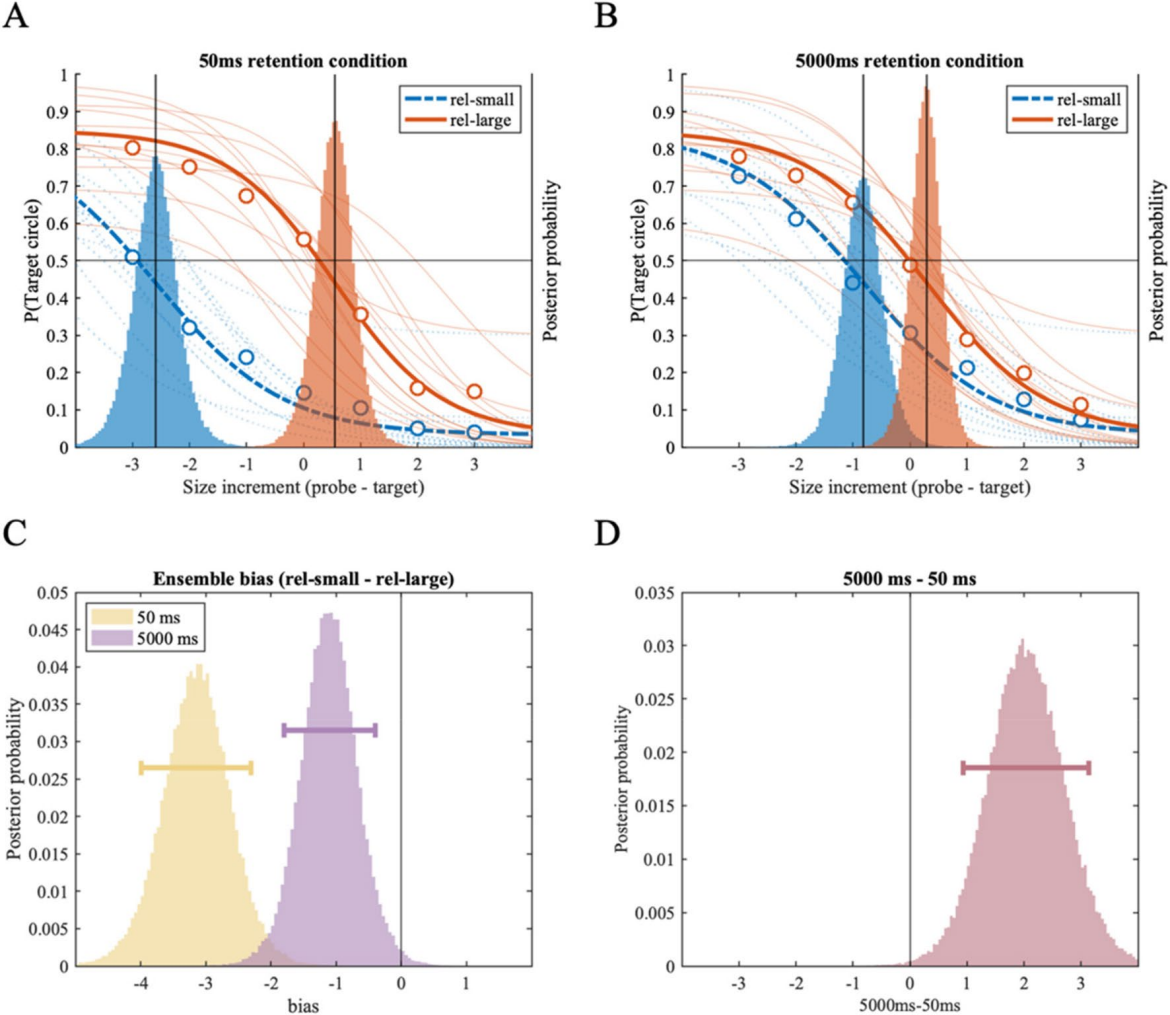
Results of Experiment 3. Two panels on the top (**A:** the *50-ms retention period,* **B:** the *5,000-ms retention period*) show fitted psychometric functions on the probability of reporting the target circle as larger (y-axis), as a function of size increment of the probe circle compared to the target circle (x-axis). Size increments on the x-axis correspond to the discrete set of 19 stimulus sizes used in the experiment; each increment is an 8% increase in perceived size (see *Method* section and Table 1). Group-level (thick lines) and individual-level (thin lines) psychometric functions are plotted for the *rel-small* (dotted line) and *rel-large* (solid) conditions; corresponding with the y-axis on the left. Group-level posterior distributions of location parameter (α) are plotted as a histogram behind the psychometric functions, referenced with the y-axis on the right. (**C**) Histograms showing the effect of ensemble representation on individual size representation in the *50-ms* and *5,000-ms conditions*, respectively. Each histogram is generated by subtracting the location posterior distribution of the rel-large condition from that of the rel-small condition from A and B plots above; negative values indicate repulsion bias away from the ensemble mean size. (**D**) Histogram showing the difference between the distributions of ensemble bias in the *50-ms condition* and the *5,000-ms condition*, indicating changes in ensemble bias with additional memory delay in the *3,000-ms condition*. Horizontal error bars indicate the 89% highest density interval

In Experiment 3, we found a robust repulsion bias in both the *50-ms and 5,000-ms retention period conditions*, indicated by shifts in psychometric function between the *rel-small* and *rel-large size conditions* (Figs. 7A and 7B). Repulsion bias was confirmed in the *50-ms retention period condition* with 100% of posterior samples larger than zero (MAP = -3.11, 89% HDI [-3.99 -2.31]) and the 5,000-ms retention period condition with 99.13% posterior samples smaller than zero (MAP = -1.13, 89% HDI [-1.79 -0.40]); Fig. 7C.

Although the repulsion bias was strong in both retention period conditions, it was credibly stronger at the *50-ms retention period condition* compared to the *5,000-ms retention period condition*, indicated by 99.75% of posterior samples larger than zero (MAP = 1.98, 89% HDI [0.94 3.13]); Fig. 7D. Note that in this experiment, the two retention period conditions had identical experiment designs where only the retention period varied (50 ms vs. 5,000 ms). This result suggests that the mnemonic process does influence ensemble bias, such that repulsion bias is strongest following a very short memory delay, and is reduced (but not eliminated) after a longer delay.

## Meta-analyses across experiments

**Fig. 8:**
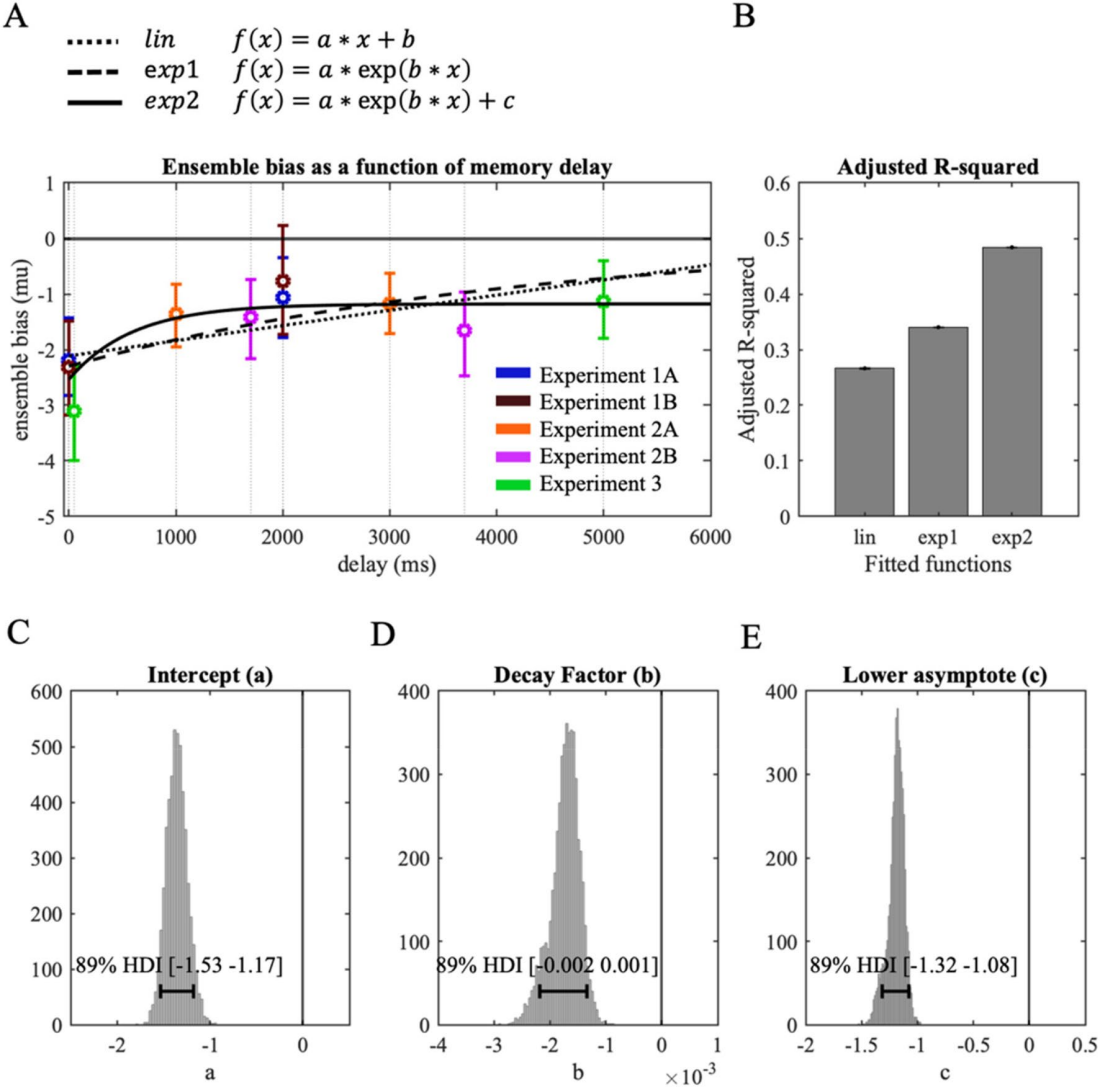
Meta-analysis result. (**A**) MAP (circles) and 89% HDI (vertical error bars) of ensemble bias (mu) from all Experiments are shown as a function of memory delay. Dotted, dashed, and solid lines respectively show fitted linear, exponential, and exponential with asymptote functions. (**B**) Adjusted R-squared measures were compared across fitted functions. The R-squared value was highest for the exponential function with an asymptote parameter, and therefore, we analyzed the (**C**) intercept, (**D**) decaying factor, and (**E**) asymptote of the exponential function. Horizontal error bars indicate 89% highest density interval of the sample distribution

To provide a better understanding of the temporal changes in ensemble bias across the memory retention periods tested in the different experiments above, we performed a meta-analysis by fitting posterior distributions of ensemble bias obtained from all five experiments (Fig. 8A).

First, we retrieved the posterior distributions of ensemble bias (panel C in Figs. 2, 3, 5, 6, and 7) estimated across the different delay conditions from all Experiments. Figure 8A shows the maximum a posteriori estimation (MAP; circular marks) and 89% highest density interval (HDI, vertical error bars) of ensemble bias obtained from each experiment. The x-axis indicates the retention period that the target circle was maintained in memory until the probe circle appeared. The retention period for the *P condition* in Experiments 1A and 1B was considered as 0 ms, and the retention periods for Experiment 2B were considered as 1,700 ms and 3,700 ms, combining the initial retention period (1,000 ms or 3,000 ms), post-cue duration (200 ms), and additional delay following the post-cue (500 ms). Visual inspection of Fig. 8A suggests a pattern where ensemble bias was strongest in the P (0 ms) and 50-ms memory delay conditions, rapidly reduced, and remained fairly stable in magnitude between 1,000-ms and 5,000-ms memory delays.

To quantitatively assess how the ensemble bias changes across memory delay, we performed an analysis where for each of 5,000 iterations we extracted 12 random samples (matching the participant sample size) from a posterior distribution of each experiment and memory delay condition, then fitted three different types of functions to the sampled data: linear function, exponential function, and exponential function with asymptote parameter. Figure 8A shows the three fitted functions constructed with MAP values of 5,000 estimated parameters for each function.

First, we compared the goodness-of-fit between functions by calculating adjusted R-squared values, which adjust for the number of predictors included in the model (Miles, 2005). As shown in Fig. 8B, we found the largest adjusted R-squared in the exponential function with an asymptote parameter (exp2), followed by the exponential (exp1) and the linear function (lin). A better fit with exponential functions compared to the linear decay function suggests the non-linear reduction in repulsive ensemble bias over the retention period. Moreover, adding an asymptote parameter to the exponential function robustly increased goodness-of-fit, suggesting that the repulsion bias was not eliminated, but rather remained stable at a reduced magnitude following a rapid decrement.

Next, we analyzed the sample distributions of the intercept (a), decaying parameter (b), and lower asymptote (c) parameter of this best-fitting function, to quantify how the ensemble bias changes across the memory retention period. The intercept (a) of the function indicates the direction and magnitude of ensemble bias at the early timepoints (minimum memory delay) relative to the asymptote. As shown in Fig. 8C, the distribution of the estimated intercept was located below zero (MAP = -1.37, 89% HDI [-1.53 -1.17]), indicating that the ensemble representation influenced the representation of individual items, in a form of repulsion bias, even when there was limited time for the mnemonic process. Next, the distribution of the decaying factor estimates (b) was credibly different from zero (Fig. 8D; MAP = -0.0017, 89% HDI [-0.002 -0.001]), suggesting a non-linear change in repulsion bias across memory delay. Lastly, the distribution of the asymptote parameter (c) was also credibly smaller than zero (Fig. 8E; MAP = -1.17, 89% HDI [-1.32 -1.08]), consistent with a lingering repulsive ensemble bias that persists across the longer retention periods.

## Discussion

Concurrent objects in the visual world are not processed independently from each other. Instead, the representation of surrounding visual information is known to influence the representation of task-relevant items. For example, a circle appears to be larger when surrounded by smaller circles (Ebbinghaus illusion; Ebbinghaus, 1902; Roberts et al., 2005), or the remembered color of an individual item is either attracted toward or repulsed away from the colors of simultaneously presented memory items (Chunharas et al., 2022; Scotti et al., 2021). In the current study, we focused on a specific type of bias, the effect of group-level ensemble representation on the item-level representation of an individual item’s size (Brady & Alvarez, 2011; Hsieh et al., 2021; Utochkin & Brady, 2020), and attempted to tease apart the perceptual or mnemonic contributions to the observed effect. Across five experiments we found robust ensemble bias, indicated by the reported size of individual circles biased away from the mean size of simultaneously presented circles. Critically, we found the repulsion bias to be strongest when there was a minimal mnemonic process and dampened with a longer memory retention period.

The effect of ensemble representation on individual item representation supports the idea of simultaneous processing of visual information at multiple levels (Brady et al., 2011; Choi et al., 2024; Hochstein & Ahissar, 2002; Wolfe et al., 2011). Decades of studies have proposed how the complex visual environment is encoded in varying details; featural versus holistic processing (Navon, 1977), selective versus non-selective (Wolfe et al., 2011), or focused versus distributed attention modes (Baek & Chong, 2020). For instance, the dual-path model (Wolfe et al., 2011) suggested distinct pathways of visual processing working in parallel; a “nonselective” pathway extracting statistical properties of basic features or gist of a scene (e.g., layout), followed by a capacity-limited “selective” pathway responsible for processing individual objects with more details. In a similar vein, Brady and Alvarez (2011) suggested a hierarchical encoding of complex visual environments through multiple levels of abstraction. Furthermore, they found the biased representation of individual items toward the group-level information of same-colored items, indicating an interaction between item- and group-level visual information (Brady & Alvarez, 2011). Henceforth, a series of studies found how the visual system uses various grouping cues (e.g., similarity, proximity) to cluster multiple items so that the visual feature (e.g., orientation, size, color, emotion) of an individual item is biased relative to the summary statistics of an affiliated group (Corbett, 2017; Son et al., 2020; Yildirim et al., 2018).

What is the advantage of encoding and integrating visual information in a hierarchical manner? While real-life scene image is complex, it contains structure, regularity, and redundancy (Geiser, 2008; Kaiser et al., 2019; Kersten, 1987). Thus, the processing of individual objects could benefit from global-level visual information of a scene (Brandman & Peelen, 2017; Davenport & Potter, 2004; Furtak et al., 2022; Lauer et al., 2018). Indeed, the recognition of degraded objects was enhanced when presented with their original scene context, compared to when presented in isolation (Brandman & Peelen, 2017). Likewise, the adaptive framework of visual working memory distortion explains inter-item distortion (e.g., attraction or repulsion) as a reflection of the optimal visual system that strategically makes use of surrounding visual information to reduce error (Chunharas et al., 2022). For example, the more uncertain the representation of individual items is, the greater it relies on information about the entire set, leading to attraction bias. In contrast, if similar items are encoded veridically, the representation of items will repel from each other to increase distinctiveness between items.

## Repulsion bias away from the mean size of a group

The ensemble bias reported in previous studies is often characterized by attraction bias toward group ensemble representation (Brady & Alvarez, 2011; Corbett, 2017; Son et al., 2020; Utochkin & Brady, 2020; Yildirim et al., 2018). For example, Utochkin and Brady (2020) presented four oriented isosceles triangles and found the representation of individual orientations to be substantially biased toward the mean orientation. However, in the current study, we found robust repulsion bias away from the group ensemble representation. The adaptive framework (Chunharas et al., 2022) suggests that repulsion bias serves to increase distinctiveness between confusable items and reduce error. Since ensemble representation exerts different influence on individual item’s representation depending on the number of items (Chunharas et al., 2022), memory load (Lively et al., 2021), or item similarity (Chunharas et al., 2022; Son et al., 2020; Utochkin & Brady, 2020), the observed repulsion bias may be specific to the current design of procedure or stimuli.

Interestingly, Rafiei et al. (2021) recently showed that the orientation judgment of a line is biased away from an ensemble of to-be-ignored items (i.e., distractors in visual search stimuli). One of the noticeable manipulations of the current study is that the smallest or largest (or second-to-smallest/second-to-largest in Experiment 1B) circle was always cued among a set. With such manipulation, it is possible that subjects strategically searched for, and selected, extreme values (e.g., either the smallest or largest circle), while ignoring items closer to the mean size. Ignoring circles closer to the mean size may have resulted in the repulsion bias we observed (Rafiei et al., 2021). However, considering that we also found repulsion bias even when a circle with a non-extreme size was cued to be reported (OSM Fig. 2), it is unclear if this actually contributes to the mechanism underlying the observed ensemble bias.

Additionally, it is plausible that the interdependency between low-level visual information is responsible for the observed repulsion bias at the early encoding phase. For example, repulsion bias for multiple circles with varying sizes resembles the Ebbinghaus illusion, where the object surrounded by smaller/larger objects appears to be larger/smaller (﻿Ebbinghaus, 1902). While earlier studies suggested the contrast between the size of adjacent items to be responsible for the Ebbinghaus illusion (The size contrast theory: Coren & Miller, 1974), recent investigations are in favor of the low-level interaction between visual contours (The contour interaction theory: Sherman & Chouinard, 2016; Todorović & Jovanović, 2018). Although the reference circles were not spatially *surrounding* the target circle in our experiment design, the low-level interaction between outlined circles may be responsible for the perceptual repulsion bias. It is also possible that our results could reflect a combination of strong perceptual repulsion bias, followed by weak attractive memory bias, which is discussed further below.

## Reduced repulsion *bias* across memory maintenance

In addition to the strongest repulsion bias with minimal memory delay, we found reduced repulsion bias with a longer memory retention period (Fig. 8). The reduction of repulsion was unexpected based on previous studies where repulsion bias increased with a longer delay (Chunharas et al., 2022; Scotti et al., 2021). We propose a few possible explanations. Possibly, the discrepancy is due to there being distinct types of repulsive bias. While the previous studies noted above presented two items to investigate inter-item interactions in working memory, our stimuli consisted of four or more circles to facilitate the processing of ensemble representation of many items (e.g., mean size) to investigate how mean ensemble representations interact with individual item representations. It is also possible that in the current study, the size representations of individual items were encoded with lower fidelity than in the prior studies and became noisier in memory, resulting in reduced repulsion bias after a memory retention period.

The results could reflect an interpretation where this sort of ensemble bias is primarily a perceptual-level effect, which simply fades somewhat upon being encoded into longer working memory. Alternatively, it is possible that the smaller repulsion bias could imply a delayed contribution of an opposing attraction bias toward the mean size of a group (Hsieh et al., 2021). In other words, decreasing repulsion bias with a memory delay may reflect either a passive weakening of the repulsion bias or an increase in a competing attraction bias effect. As individual size representation becomes noisier following a longer retention period, the visual system may rely more on the ensemble properties (Chunharas et al., 2022; Lively et al., 2021; Rademaker et al., 2018), counteracting the repulsion that occurred at the early perceptual encoding phase.

Interestingly, the sudden reduction of repulsion bias at early retention delay followed by sustained repulsion bias for a longer retention period (Fig. 8E) resembles the time course of the exponential decaying function of iconic memory (Lu et al., 2005). Further investigation of the relationship between memory decay and ensemble bias offers an intriguing direction for future study toward a better understanding of the broader contributions of perceptual and mnemonic processing.

## Was the set size sufficient to extract the mean size?

To ensure the number of circles is within known working memory capacity (Luck & Vogel, 1997), we presented four items as a reference set. However, a potential concern was whether the number of circles presented for the reference set is sufficient to extract ensemble representation in the form of distributional properties (e.g., mean, deviation). Previous literature has used four items to study ensemble perception, suggesting four items are enough to extract mean properties of low-level visual information (Allik et al., 2014; Haberman et al., 2015; Utochkin & Brady, 2020). Meanwhile, previous studies have also shown better extraction of the distributional property of low-level visual information with a larger set size: mean size (Robitaille & Harris, 2011), orientation (Chetverikov et al., 2017; Robitaille & Harris, 2011), or color (Maule & Franklin, 2015). When we presented 12 circles as a reference set for better extraction of ensemble representation (Experiment 1B), we found a consistent result compared with when we presented four circles, suggesting that the observed pattern of results is not specific to a small number of items presented.

## Focused versus distributed mode of attention

In Experiment 2B (and the supplementary experiment), we facilitated a distributed mode of attention across multiple potential target items and found the same pattern of results compared to the experiment design that facilitated focused attention on a single target. According to previous studies, the ensemble representation of multiple items is better encoded under the distributed mode of attention (Baek & Chong, 2020; Chong & Treisman, 2005). Moreover, individual orientations (Utochkin & Brady, 2020) or mean size of a subset of stimuli (Yildirim et al., 2018) relied more on the summary statistics of entire stimuli when cued after stimuli presentation (post-cue) compared to when cued before (pre-cue). To examine whether different modes of attention could change the pattern of observed ensemble bias, Experiment 2B post-cued a target circle in a set of circles to facilitate distributed attention. Because four circles were encoded simultaneously with distributed attention and maintained until the target circle was post-cued, the fidelity of representations and sensitivity for the size comparison task was expected to decrease in Experiment 2B. Indeed, in Experiment 2B, we found a shallower slope in the fitted psychometric functions compared to that of Experiment 2A (89% HDI [0.10 0.57], pooled across conditions). Despite the effect of distributed attention on size comparison task performance, it did not change the magnitude of the repulsion bias nor the effect of the additional memory retention period (Figs. 5 and 6), underscoring the robustness of these results.

## Asymmetricity

Ensemble bias was defined as a difference between the *rel-small* and *rel-large conditions*. From a theoretical perspective, because many factors, including individual differences, can influence overall biases to respond larger or smaller in different contexts, it was critical to define ensemble bias as the relative difference between these two otherwise equated conditions. However, it is intriguing that in most of the experiments, the ensemble bias seemed to be largely driven by deviated location parameters in the *rel-small condition*. In other words, the size representation of the target circle was more likely to be biased away from the mean size of a group when it was the smallest among a group, rather than the largest.

Such asymmetry may be attributed to a non-linear relationship between the physical and perceived size of visual objects. Although we accounted for the non-linearity by converting physical size into perceived size by the power function suggested by Teghtsoonian (1965), there may be individual differences that must be accounted for when converting between physical and perceived size. Next, it remains unclear whether humans perceive the size of stimuli based on the diameter, area, or some unknown measures (Raidvee et al., 2020; Solomon, 2021). In the current study, we focused on the perceived area when generating a set of circle sizes with an 8% increment (5% in Experiment 1B; Table 1). Moreover, subjects were simply instructed to report which circle was “larger,” without an explicit definition.

Alternatively, the asymmetry may occur because attention is naturally guided toward a larger item among a set (Proulx, 2010; Proulx & Green, 2011). When attention was directed toward the largest item, that item might be encoded most veridically. As a result, the ensemble representation of concurrent items may not have as much effect in the *rel-large condition* compared to the *rel-small condition*. In addition, it is also possible that attended items are weighted more when computing the mean size of multiple items, resulting in overestimated mean size (Choi & Chong, 2020; De Fockert & Marchant, 2008; Kanaya et al., 2018). The overestimated mean size will be more distanced from smaller circles among a set, which might result in a greater influence of mean size in the *rel-small condition*. Lastly, the requirement to identify the larger item, but not the smaller item, could be another potential source of the asymmetry. Indeed, the additional experiment, in which subjects were asked to directly report the size of the cued circle via a continuous report method, did not show the asymmetry found in the main experiments with the 2AFC task (OSM Fig. 2). Given these different possibilities for the asymmetry, we are careful not to over-interpret the asymmetry, and emphasize the ensemble bias measure defined as the relative difference between the *rel-small* and *rel-large conditions*.

## Conclusion

To conclude, the current study investigated the perceptual versus mnemonic source of the effect of ensemble representation on individual item size representation. We designed psychophysical experiments that systematically manipulated the involvement of perceptual and mnemonic processes to varying degrees. We found a strong repulsion bias away from the mean size of a group at the early encoding phase. By comparing ensemble bias across a wide range of delay period conditions, we found that the repulsion bias is reduced within a short retention period, and then quickly asymptotes into a stable bias that persists up to 5 s in memory. These results suggest a complex interplay between perceptual and mnemonic processes underlying observed ensemble bias.

## Supplementary information

Below is the link to the electronic supplementary material.

Supplementary file1 (DOCX 474 KB)

## Data Availability

Raw data for all experiments have been made publicly available in the Open Science Framework (https://osf.io/vkx5h/).

## Code availability

MATLAB scripts to run experiments and analyze data for all experiments have been made publicly available in the Open Science Framework (https://osf.io/vkx5h/).
