# Supplementary material for: The perceptual and mnemonic effects of ensemble representation on individual size representation
Source: Atten Percept Psychophys. 2024 Oct 9;86(8):2740–60. doi: 10.3758/s13414-024-02963-x (PMC11652647; doi:10.3758/s13414-024-02963-x)
Supplement: Supplementary file 1 — Supplementary file1 (DOCX 474 KB) [file 13414_2024_2963_MOESM1_ESM.docx]

**
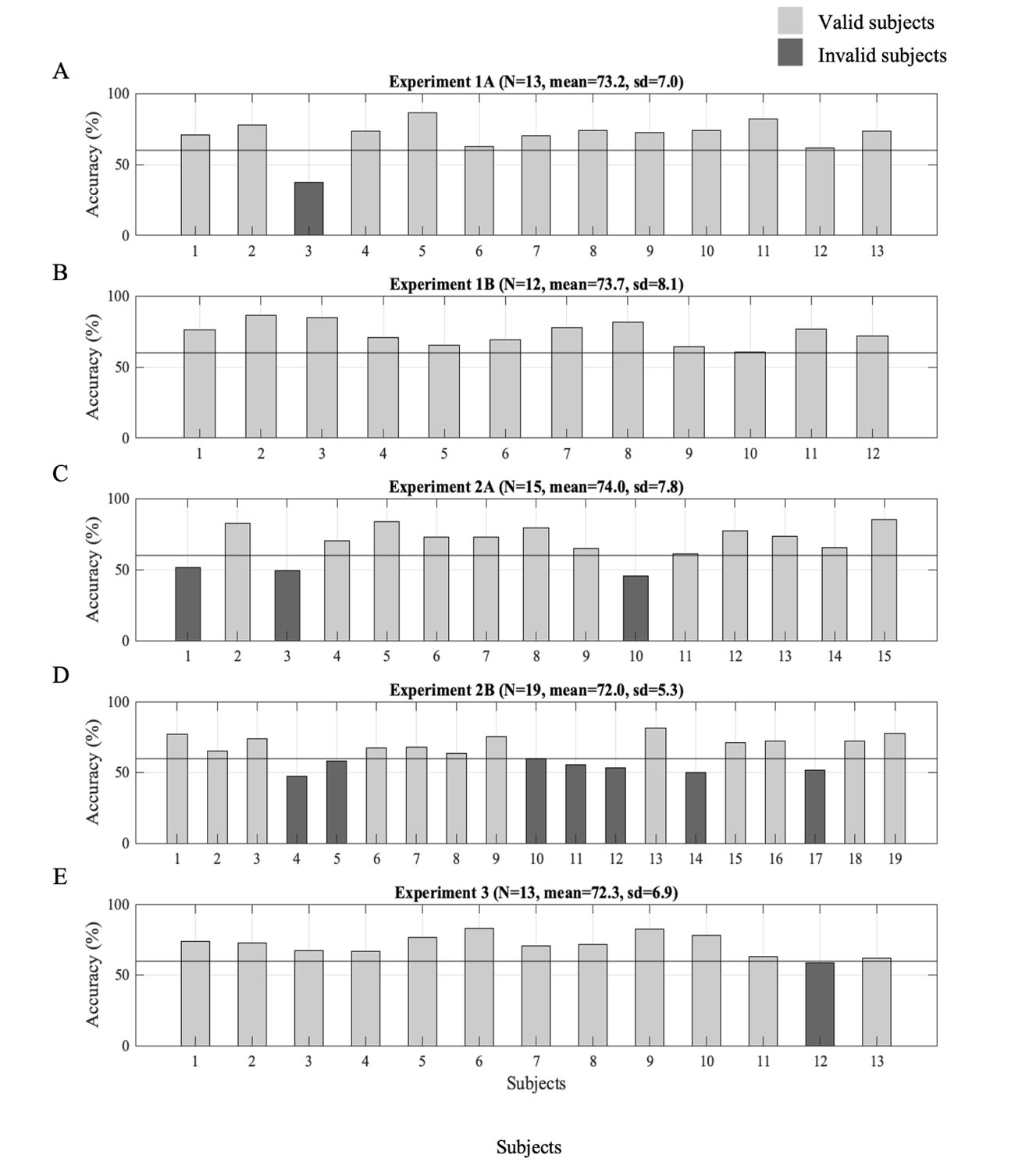
Supplementary Materials**

***Supplementary Figure 1. The accuracy results of all Experiments***

The accuracy (%) for all experiments using the size comparison task was calculated after excluding trials where the size of two circles was identical. Subjects with lower than 60% accuracy (horizontal solid line) were excluded from the analysis (dark gray).

***
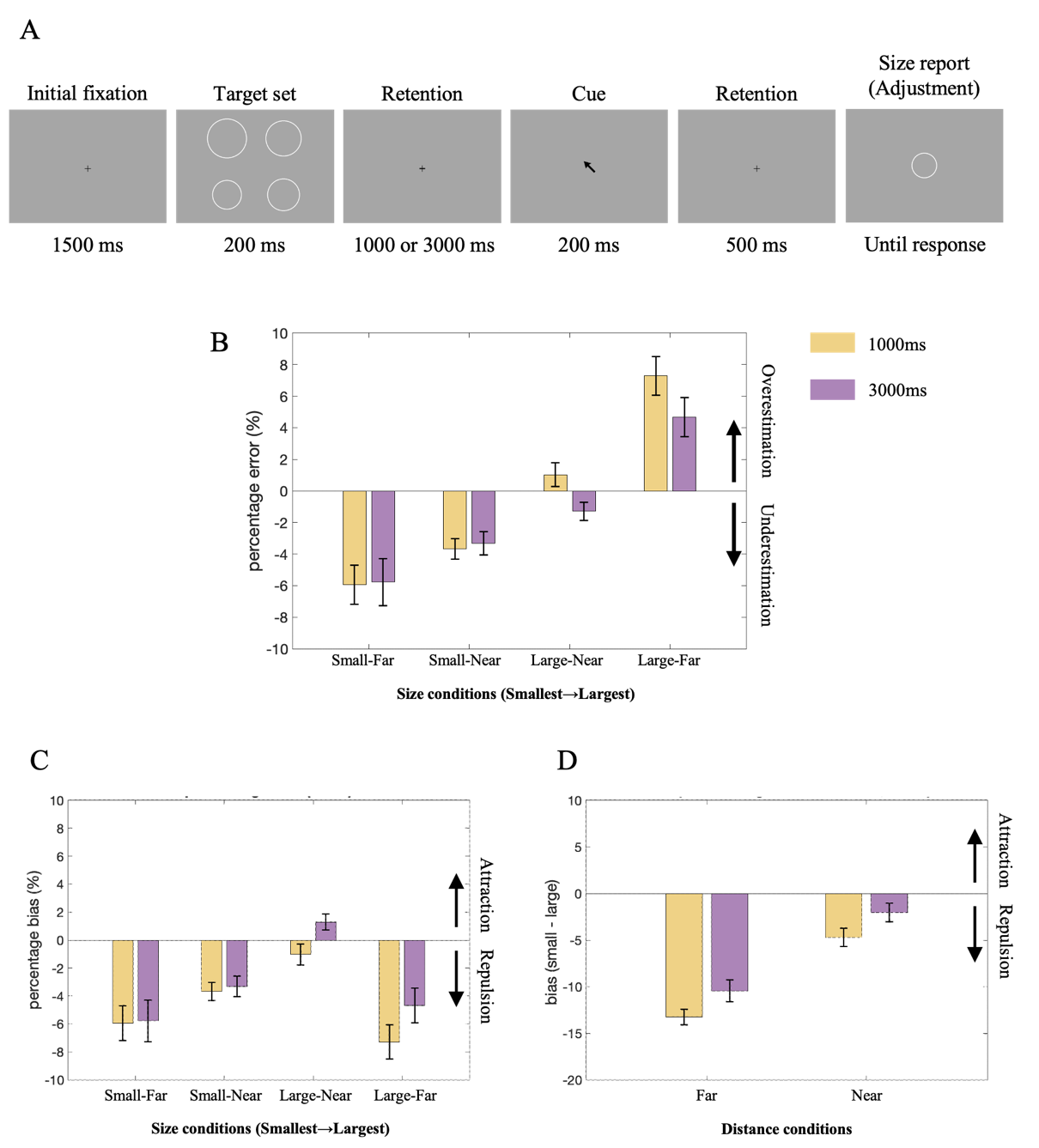
***

***Supplementary Figure 2. Supplemental Experiment using continuous report paradigm***

The experiments described in the main manuscript employed a 2AFC size comparison task and psychometric function approach. To confirm if the repulsion bias findings generalize to a different experiment paradigm and to examine ensemble bias among all members of a set, we conducted an additional experiment using a continuous report task. The experiment was analogous to Experiment 2B in the main text, and was pre-registered at OSF: https://osf.io/vkx5h/. 27 subjects (18 women, 9 men, 0 non-binary; mean_age_ = 20.48, sd_age_ = 4.09) completed the experiment. Note that we collected three more subjects than the pre-registered sample size (N=24). However, the pattern and significance of the results remained unchanged without the last three subjects.

(A) Each trial started with briefly presenting four white circles of varying sizes, followed by either a 1,000 ms or 3,000 ms retention period (*delay condition*). Then, a post-cue arrow was presented, pointing at the spatial location of the target circle to be reported. After another 500 ms retention period, a single probe circle appeared at the center of the display, and subjects were asked to report the size of the target circle by adjusting the size of the probe circle (i.e., continuous adjustment method). All four circles from the smallest to the largest circle among a target set could be the target circle. The four circles on each trial were categorized relative to the mean size of the set, in terms of relative size direction (*smaller* or *larger*) and distance (*near* or *far*) from the mean ensemble size. Thus the four circles from the smallest to largest circle were labeled as *small-far, small-near, large-near, and large-far* size conditions.

(B) The reported size (radius in pixels) for each condition, was converted into *percentage error* (reported size – actual size / actual size $\times$ 100) relative to the mean size of a group (Supplementary Figure 2B). Analogous to the results of the main experiments, the size of the smallest circle in the set (*small-far*) was underestimated, and the size of the largest circle in the set (*large-far*) was overestimated, reflecting repulsive ensemble bias away from the mean size of a set. To examine the ensemble bias, we performed two lines of analysis: a preregistered analysis (C) and an analysis analogous to the main experiments (D).

(C) *Pre-registered analyses.* We calculated *percentage bias* by flipping the sign of the *percentage error* in the *large-near* and *large-far* size conditions so that positive and negative values respectively indicate attraction and repulsion bias (Supplementary Figure 2C). First, we performed a one-sample *t*-test for each condition after pooling across delay conditions. The repulsion bias measured in this way was statistically significant only when the largest circle among the set was reported (*large-far*; *t*(26)=-2.423, *p*=.023, *d’*=-0.47. *BF*_10_=2.36). Next, we performed a 2 (*small* & *large*) $\times$ 2 (*near* & *far*) $\times$ 2 (*1000 ms* & *3000 m*s) repeated measures ANOVA to examine the ensemble bias across size and delay conditions. The main effect of distance from ensemble mean was significant (*F*(1,26)=24.98, *p*<.001, $\eta_{p}^{2}$=0.49, *BF*_incl_=1.67), indicating a larger ensemble bias (i.e., repulsion bias) for the target circles further away from the mean size of a group. The main effect of size direction was not significant (*F*(1,26)=0.12, *p*=.729, $\eta_{p}^{2}$=0.005, *BF*_incl_=0.23). The main effect of memory delay was significant (*F*(1,26)=11.43, *p*=.002, $\eta_{p}^{2}$=0.305, *BF*_incl_=0.19), suggesting a greater bias for the 1,000 ms compared to the 3,000 ms condition. Note, however, that this effect was somewhat inconclusive because frequentist and Bayesian statistics supported alternative and null hypothesis, respectively. There was a similarly inconclusive interaction effect between the size and distance condition (*F*(1,26)=22.30, *p*<.001, $\eta_{p}^{2}$=0.462, *BF*_incl_=0.23). We found no significant size $\times$ delay interaction (*F*(1,26)=3.35, *p*=.079, $\eta_{p}^{2}$=0.11, *BF*_incl_=0.24), distance $\times$ delay interaction (*F*(1,26)=0.01, *p*=.933, $\eta_{p}^{2}$<0.001, *BF*_incl_=0.19), three-way interaction (*F*(1,26)=0.09, *p*=.771, $\eta_{p}^{2}$=0.00, *BF*_incl_=0.25).

(D) To provide a more direct comparison to the experiments in the main manuscript, we conducted an additional set of analyses quantifying repulsion bias as the relative difference between the small and large size conditions, which is arguably a more sensitive, robust, and appropriate measure (see main text Discussion). In the main manuscript, we quantified the bias by calculating a difference in horizontal locations of psychometric functions between *the rel-small* and *rel-large* conditions. The analogous approach here would be quantifying the ensemble bias by calculating the difference in the *percentage error* (unflipped sign, panel B) of the large size condition from that of the small size condition. Supplementary Figure 2D plots the bias calculated this way as a function of distance from the ensemble mean and retention delay. In all four conditions, we found a significant repulsion bias away from the mean size (one-sample *t*-tests vs zero: all *p*s<.05, *BF*_10_s>1.4). Paired t-tests also revealed a significant effect of memory delay, with repulsion bias significantly greater after the 1000 ms retention delay compared to the 3000 ms condition in both the Far-distance (*t*(26)=-2.46, *p*=.021, *d’*=-0.47, *BF*_10_=2.52) and Near-distance (*t*(26)=-2.78, *p*=.01, *d’*=-0.54, *BF*_10_=4.71).

Combined, the supplemental experiment demonstrated robust repulsion bias away from the mean size under distributed mode of attention, using a continuous report paradigm. We found repulsion bias was consistently stronger for the more extreme-sized circles in a set (further from the ensemble mean), but when quantified with the more sensitive difference measure, significant repulsion bias was found for all four circles among a target set. Similar to main Experiment 2B, we found significant repulsion bias at both 1,000 ms and 3.000 ms delays. Here we further found evidence suggesting decreased repulsion bias with the 3,000 ms memory retention period compared to the 1,000 ms memory retention period; although the difference between these two delays was not present in main Experiment 2B, the directionality of the delay effect corresponds with the overall pattern of reduction in ensemble bias across the memory retention period (Figure 8. Meta-analysis).
